# Physical Violence and Scapegoating Within the Family: An Exploration of Biblical Texts and Contemporary Psychology

**DOI:** 10.1007/s10943-023-01818-3

**Published:** 2023-05-11

**Authors:** Saša Poljak Lukek, Tanja Pate, Christian Gostečnik

**Affiliations:** 1 Department of Marital and Family Therapy and Psychology and Sociology of Religion, Faculty of Theology, University of Ljubljana, Ljubljana, Slovenia; 2 Slovenia and Franciscian Family Institute, Ljubljana, Slovenia

**Keywords:** Physical punishment, Scapegoat, Sacrifice, Redemption, Family

## Abstract

To understand physical violence in the family, it is important to define the role of the victim. The term “scapegoat” is a universal anthropological concept, often used in sociological theories, where a certain group of people and/or minorities are often victimized or blamed (e.g., social ills). We may note that the phenomenon of scapegoating is most clearly expressed in the Bible. Therefore, we will use relevant biblical texts that refer to parental use of corporal punishment in which a child is scapegoated and/or victimized by parental violence. In this sense, the Bible is the most profound explanation and manifestation of the cultural, social, and especially religious development of humanity. At the same time, the concept of scapegoating is also demonstrated in psychology and therapy, where it also serves as a basis for understanding, for example, physical violence in the family, and where it is also crucial to define the role of the victim. In this article, therefore, we will explain the biblical background of this concept and highlight two basic dynamics of violence against children in the family: when the child is the “scapegoat” for unresolved tensions in the family and when the child becomes the “sacrifice” or victim of the dysregulated emotional response of his or her parents.

## Introduction

Physical violence is a burning issue in family parenting today. According to many professionals, corporal punishment is clearly unjustified (Gershoff & Grogan-Kaylor, 2016), but it is still recurrent (DuRivage et al., 2015). Research shows that spirituality can be an important protective factor for the mental health of an adult who has experienced physical violence during childhood (Westerfield & Doolittle, 2021) and that it is positively associated with the emergence of family well-being (Ming, 2014). Religiosity also shapes the relationship between children and their parents, the parental experience of the child in the family and parenting practices (Petro et al., 2017). In a meta-analysis of the relationship between religiosity and parenting, Petro et al. (2017) assert that while there are tendencies for religious parents to have a more authoritative parenting style, research does not show a relationship between a particular parenting style and parents’ religious beliefs. On the other hand, one study suggests that religious beliefs can sometimes lead to regressive behavior, damage family relationships and harm children’s growth and development if they have become a source of conflict in a family and as well lead to the application of an authoritarian parenting style (Riany et al, 2017). Recent study which examined religious beliefs and parenting styles in predicting children’s behavioral problems, as well as investigated the mediating role of digital literacy to explain this relationship, has found that religious beliefs can affect authoritarian parenting styles and negatively influence digital literacy (Purnama et al, 2022). While conservative religious practices are believed to be associated with increased approval of corporal punishment of children, parental religiosity is believed to lead to positive parenting and better child development outcomes (Mahoney et al, 2001), especially when parenting styles that are emotionally responsive, often involved in children’s activities, and democratic show a positive relationship to firm parental religious beliefs (Williams et al, 2019). The parental experience of the child is the most important factor in shaping the parental role, and the parent’s values and religiosity have a significant influence.

Severe parenting is often associated with corporal punishment in upbringing. A recent study on the use of corporal punishment in Europe showed that 91.1% of parents irregularly “hit, spank or slap” their child when the child misbehaves and that 8.9% of parents do so frequently; the likelihood of parental corporal punishment is 1.7 times higher in countries where corporal punishment is not regulated (prohibited) by law (DuRivage et al., 2015). According to an international study, 63% of parents in developing countries report using corporal punishment in the past month (results range from 28% in Bosnia and Herzegovina to 84% in Jamaica) (Lansford & Deater-Deckard, 2012). Meanwhile, a study of a representative group of the American population showed that 57% of mothers and 40% of fathers physically punish (spank) three year olds and that 52% of mothers and 33% of fathers physically punish (spank) five year olds (MacKenzie et al, 2012). Physical punishment is associated with increased aggression in the child, increased antisocial behavior, more externalized and internalized problems, increased psychological problems, and a more negative relationship parents. In addition, these children may be found to have a lower capacity for moral judgment, along with lower cognitive ability and self-esteem (Gershoff & Grogan-Kaylor, 2016). These data lead us to conclude that zero tolerance for corporal punishment is necessary in parenting and for healthy psychological development of each individual. Parents need support and guidance in more developmental parenting methods where their religiosity or spiritual values play an important role. Through the biblical description of the parental relationship with their children and their respective roles, we aim to show both the conservative concept of the child as scapegoat and sacrificial lamb and ways of redemption and reconciliation through parenting.

By combining biblical texts and the modern psychological understanding of the parental relationship, we will illustrate the dynamics of domestic violence. Through the notion of the child as scapegoat and sacrificial lamb, we will portray the subordinate role of the child in the family dynamic and define parental responsibility in parenting. In order to understand family dynamics, it is important to accept the child’s unconditional loyalty to family relationships (Winnicott, 1965) and the often unintentional transference of parental psychological content into the child subsystem (Schore, 2016). However, reconciliation and redemption are recognized in the action of the servant of the Lord, when he himself becomes the offering and sacrifice for the sins of the people. A child then becomes a value and deserves respect. In familial relationships, however, redemption consists of the parents’ ability to take responsibility for their own experiences as they enter into other relationships through vulnerability and sincerity (Firestone, 2000) or transform their inner experiences and consequently the inner experiences of their offspring (Siegel & Solomon, 2013; Stern et al., 2010). Breaking free from old patterns, taking responsibility, respecting fellow human beings, and reconciliation allow for breaking the cycle of violence that is passed down through generations in the family system.

## The Child as a Scapegoat

In this section, we will first present relevant biblical passages that relate to raising and educating children to illustrate the ancient socio-historical perspective and principles or patterns regarding the perception of violence and corporal punishment within the family, and then we will address the dynamics of scapegoating. These themes are probably most clearly expressed in the book of Proverbs: *“Whoever spares the rod hates their children, but the one who loves their children is careful to discipline them« (Proverbs 13:24).» Blows and wounds scrub away evil, and beatings purge the inmost being«. (Proverbs 20:30) The sluggard says, “There’s a lion outside! I’ll be killed in the public square!” (Proverbs 22:15).» Do not withhold discipline from a child; if you punish them with the rod, they will not die. Punish them with the rod and save them from death« (Proverbs 23:13–14).» A rod and a reprimand impart wisdom, but a child left undisciplined disgraces its mother« (Proverbs 29:15).* Using these parenting criteria, we can illustrate a very significant biblical phenomenon called scapegoating, which highlights not only a religious concept but also a social dimension, especially in biblical times. We can see that religious and social values and laws influenced each other. Of course, we can also make clear that, on the other hand, social life in biblical times was also very strongly and predominantly permeated by religious rituals and practices.

In this sense, we can also note that the dynamic of scapegoating is not unique to biblical or Christian terminology, but is universal and is also used in modern parenting. In addition to this parenting dynamic, we can note that the scapegoat dynamic is also evident in political social unrest, for example, and as a result or outcome we can think of moral heroes as victims of social revolutions of all kinds.

### Child as a Sacrifice for the Sins of their Parents

Since we are guided by biblical texts, the first thing we can point out is that the scapegoat in biblical terminology represents a proprietary sacrifice for the sins of the individual:

And he shall take the two goats and put them in front of the LORD at the door of the tabernacle of the congregation. And Aaron shall cast lots over the two goats; the one lot for the LORD and the other lot for the scapegoat. And Aaron shall bring the goat on which the LORD’s lot fell, and offer it for a sin offering. And the goat on which the lot fell to become the scapegoat shall be brought alive before the LORD to make atonement with him, and to make him go into the wilderness as a scapegoat. (Leviticus 16: 7–10)

The goat is sacrificed and banished to atone for all the sins of mankind. In a family setting, children may similarly become such scapegoats to atone for all the sins of their parents, which the parents cannot bear or have not repented of. The child becomes the bearer of unresolved conflicts, deep unconscious emotions, and the unfulfilled dreams and projects of the parents. The child, who is loyal to his or her parents (Winnicott, 1965), has no choice but to literally absorb all these parental contents and, having assumed the role of scapegoat, has no real chance to defend him- or herself. Moreover, through inappropriate reactions, misbehavior and inappropriate emotional responses, the child happens to facilitate the parental channeling of their own deep wounds through inappropriate punishment or even hostile behavior toward the child.

### Atonement as a Metaphor for Scapegoat

The theological use of the term “atonement,” which refers to a cluster of ideas in the Old Testament that focus on cleansing from uncleanness, also includes the idea of a scapegoat (what must be done to prevent God from leaving the temple), and the New Testament ideas that “*Christ as a scapegoat died for our sins*” (1 Corinthians 15:3) and that “*we were reconciled to God through the death of His Son*” (Romans 5:10), which we will discuss further later. In English translations of the Old Testament, “make atonement” is usually translated “kipper,” the verb for the ritual removal of impurities from the temple or sanctuary, accomplished by sprinkling certain temple furnishings with the blood of the “purification offering” or “sin offering” on particular Temple furnishings. *Kipper* occurs most frequently, but not exclusively, in sacrificial texts. In one passage (Leviticus 16:10), *kipper* is also performed over the scapegoat. Thus, scholarly discussions of atonement in the Old Testament focus on the sacrificial and scapegoating rituals, but also deal with the procedure for paying restitution, for which the word *kopher*  (related to *kipper*) is used. The most important day in the ancient Jewish liturgical calendar was Yom Kippur, the Day of Atonement, on which the highest sacrificial rituals of the year were performed, and the only day of the year on which the scapegoat rite was performed. Atonement is further explained and expressed in the New Testament through the metaphors of sacrifice, scapegoat, and redemption to illustrate the significance of Christ’s death. We can assert that Apostle Paul is the primary source of these soteriological metaphors, which also appear in the other epistles and in Revelation. We can also note that atonement metaphors appear in the Gospels, possibly in the Lord’s Supper and in the ransom saying (Mark 10:45). We can also see that the atonement in the Old Testament is mainly concerned with the cleansing the temple (the house of the Godhead), not with soteriology. In the New Testament, on the other hand, the atonement is central to the soteriological metaphors in Paul’s letters, the deutero-Pauline letters, the letter to the Hebrews, First Peter, First John, and Revelation.

In other words, the Bible tells us that all sins will be atoned for and the covenant with God restored once the people have transferred all their sins to the scapegoat and the goat is banished into the wilderness. Thus, in Genesis we find a goat among the animals that Abraham sacrifices to God *for the covenant* (Gen. 15:9); the goat is *payment* Laban makes to Jacob for his services (Gen. 30:35); it is also Jacob’s *gift to his brother Esau* (Gen. 32:14) and *a token of redemption* (Gen. 38:17.20.23). In Genesis the goat is the animal sacrificed to atone for a sin. When a prince sins, he is to bring a goat, place a hand on the goat’s head, and offer the goat as an *offering to atone for a sin* (Leviticus 4: 22–24). Moses instructs Aron that the people are to *offer the ram as a sacrifice for sin* (Leviticus 9: 3). Moses was particularly zealous when it came to the ritual of sacrifice (Leviticus 10: 16; 23: 18), and he gave detailed instructions for the *ritual of sacrificing the he-goat for a sin* (Leviticus 10: 16; 23: 18): Aron is to *take two he-goats*, one as an offering to the Lord and the other to Asasel (*the scapegoat, T.N.)*, thus atoning for the sins of the people (Leviticus 3: 16; 5–28). Another type of sacrifice is described in Numbers: The festivities last twelve days, a prince of a certain clan is to bring his offerings on every single day, and the *goat is always mentioned as a sacrifice for sin* (Numbers 7, 16.22.28.34.40.46.52.58.64.70.76.82.87.88). Similarly, Moses gives instructions as to how the Sons of Israel were to celebrate a public service to God on certain days. There, the *offering of goats for sins* is also specifically required (Numbers 28:15, 22, 30; 29: 5, 11, 16, 22, 25, 28, 31, 34, 38).

### The Scapegoating Dynamic in the Family

Such a dynamic of sacrificing the weak is also found in families. One could say that parents transfer their unresolved psychological content to their relationship with the child, making the child the sacrificial lamb or scapegoat in the family (Cummings & Davies, 2010; Schore, 2009, 2016, 2019a, 2019b). Systemic theories therefore refer to this as an emotional triangle, where two family members are unable to relieve the stress that has accumulated in their own relationship and therefore take a third person between them (Bowen, 1978). Since the dyad is not as stable as a triad, a family system without emotional triangles cannot maintain balance. Meanwhile, the child inevitably finds him- or herself in the role of caretaker for all the tensions within the family.

The scapegoating dynamic can be similarly observed in the mechanism of transferring emotional tensions that accumulate in the marital system to the parent–child relationship. Numerous studies have confirmed a correlation between marital problems and child adjustment difficulties, as well as behavioral problems and emotional inadequacy (Cummings & Davies, 2010; Davies et al, 2009; Grych & Fincham, 1990). Studies have explained this with a correlation between marital conflict and inability to perform parental duties (Gerard et al, 2006; Kitzmann, 2000). Thus, we can assume that unresolved tensions in the marriage serve as an excuse or incentive for discordant or even violent parenting of children in the family. The child behaves in a way that gives the parents the opportunity to regulate their tensions, which they exploit because they are unable to confront the parental problems. Moreover, the results of the studies (Afifi et al. 2006; Lunkenheimer et al., 2006; Afifi et al., 2022a, 2022b) also indicate that a parent’s own history of physical abuse, emotional abuse, and spanking in childhood is associated with an increased likelihood that their child would have been spanked. These findings indicate that a parent’s adverse childhood experiences history may also tend to interpret the child’s behavior as problematic, which leads them to use more and more disordered parenting methods, and one could say that they use the child to regulate their own stress. We use the term dysregulated response when parents, due to their lack of control over their own experiences, relationship stress, and low self-esteem, use the child’s inappropriate behavior as an incentive to use punitive parenting methods (Poljak, 2009; Woodward & Fergusson, 2002) or to “sacrifice” the child to reduce their physical irritability or stress.

## The Child as Sacrificial Lamb

Especially in cases of family violence, we can speak of the child as the sacrificial lamb of the whole family. The Bible also tells a well-known traumatic story of an actual sacrifice of a child. Jephthah’s promise to God led to a dramatic sacrifice of his own daughter. The daughter had to pay for the victory of the father over his enemy.

And Jephthah vowed to the LORD and said, “If you will indeed deliver the children of Ammon into my hands by all means, then the LORD’s will be the first to come through the door of my house to me when I return in peace from the children of Ammon. I will offer them as a burnt offerings. (Judges 11: 30–31)

And Jephthah came to Mizpeh unto his house, and, behold, his daughter came out to meet him with timbrels and with dances: and she was his only child; beside her he had neither son nor daughter. And it came to pass, when he saw her, that he rent his clothes, and said, Alas, my daughter! thou hast brought me very low, and thou art one of them that trouble me: for I have opened my mouth unto the Lord, and I cannot go back. (Judges 11: 34–35)

After two months she returned to her father and he did with her what he had vowed to do. (Judges 11: 39)

The daughter of Jephthah thus became the sacrificial lamb of her father’s promise, his dreams, his desires, and his needs, and thus in a very special way also the symbol of parents who wish, or at least want, their child to fulfill their dreams in life. Parents can place an unbearable burden of their own wants and desires on their children, even if it is done with the best of wishes and intentions. Similarly, we find several instances in the Bible where a family member is made a scapegoat or sacrificial lamb to provide for a conflicted relationship on the one hand and an insatiable desire for redemption on the other. One could say that the member is chosen to reconcile the marriage or the whole family with his suffering.

### Physical Punishment as a Sacrifice of the Children

Similarly, we may observe a transfer of unfulfilled wishes and desires from parents to children, whereby the “sacrifice” of a child produces a sense of well-being in the family, in the parenting methods. Physically punishing children can be understood as a sacrifice in this sense. When they physically punish, parents may be acting with the *best of intentions,* but are placing an *unbearable burden* of their own choices on their child. Indeed, in most cases the intent of corporal punishment is not to inflict pain or injury on the child, but to limit the child’s undesirable behavior and control it with the *best of intentions* (Straus & Stewart, 1999). However, the frequent use of physical punishment, rather than helping the child to adjust, leads to problems in the child’s behavioral (Aucoin et al., 2006; Gershoff, 2002; Lynch et al., 2006), social (Elgar et al., 2018; Grogan-Kaylor, 2004; Kang, 2022) and emotional development (Aucoin et al., 2006; Harper, Brown, Arias, & Brody, 2006; Stargel et al, 2022). Since physical punishment restricts the child’s behavior without regard to specific developmental or individual needs, but parents resort to it to satisfy their own need to relieve their physical irritation (Poljak Lukek, 2019), we can say that physical punishment in modern parenting is a sacrifice of the child to achieve a sense of well-being in the family.

Aside from constraining and controlling the child’s behavior, parental physical punishment also serves as a means of physically appeasing the parent (Socolar et al, 2005). When parents are unable to control their own emotional experience of the child’s inappropriate behavior or even their presence, they may resort to physical punishment to soothe their own bodies (Gross, 2013). Such behavior is usually associated with family stress, marital conflict or parental violence, and a generally unstable family system (Woodward & Fergusson, 2002). Based on the fact that parents use physical violence more often than they approve of it (Straus & Mouradian, 1998), we can conclude that it is used impulsively, at the moment of emotional dysregulation, when they are unable to control their emotional experience. They react according to a standard mechanism of emotion regulation that, if they had been physically punished as children themselves, would have resulted in violent physical appeasement, i.e., a physical reaction (Socolar et al, 2007; Straus, 1994). This is the only way for them to appease their own bodies and, by extension, the child’s. However, continuous dysregulated modeling is known to have a detrimental effect on the child’s behavior because repeated dysregulated emotional responses are thereby internalized by the child in his or her own experience, forming an enduring pattern of emotional and behavioral maladjustment (Galambos & Costigan, 2003; Upenieks et al., 2022).

### The Sacrificial Dynamic in the Family

Of course, corporal punishment is not the only way a child can become the victim or sacrificial lamb of the family. There are other forms of punishment, not aimed exclusively at serving the needs of the child, in which we can see a similar act of sacrifice. The transfer of tensions from the parental to the child subsystem can also occur through the withdrawal of attention or through the creation of conflict between parents and their children (Gerard et al., 2006). Parents who are preoccupied with their own wants, desires, or suffering will not be able to pay attention to the child’s needs or desires or recognize the child’s suffering (Wu et al., 2022). A child who is ignored in this way has no choice but to sacrifice his or her wants, needs, and suffering in order to maintain balance in the family. Such victimization of the child against parental promises, wishes, and desires is also found in certain non-punitive parenting methods. Indeed, apart from regulating the child’s behavior, parenting also requires a response to the child’s needs (Maccoby & Martin, 1983) and the extensive use of parenting methods that do not match the child’s development and behavior may also result in the child being sacrificed to parental needs. In this case, we speak of either loose or permissive parenting, whereby the child soon feels unsafe and recognizes certain emotions as unacceptable, or uninvolved parenting, whereby children may become emotionally impoverished, confused, or anxious because there are no clear boundaries or because there is a lack of interest in their needs, desires, and emotions (Poljak Lukek, 2017).

Another relevant psychological phenomenon found in the Bible and related to the parent–child relationship is the transfer of responsibility. We read that when the father returned to his daughter, he said: “*You have humiliated me. You also belong to those who harass me*…” (Judges 11: 34). Similarly, today’s parents may feel that the child and his behavior are the direct cause of their pain and suffering. Parents often associate their stress with the child’s behavior (Neece et al, 2012), thus transferring responsibility for their own stress onto the child. As a result, any problems that arise in the family are solved by focusing on the child and his or her behavior, even though the actual source of the stress is more likely to be the parent’s choices, desires, experiences, and pain.

## The Child as the Caretaker of Intergenerational Pain and Conflict

Psychological pain and unresolved tension can also be passed down through multiple generations. In psychology, the role of the victim is defined by the establishment of the emotional triangle, emotion or affect regulation, and projective identification. Family relationships not only lead to violent reactions, but also enable the overcoming and resolution of violence. The change in affective perception in the relationships is formative for overcoming and transcending, whereby the suffering and sacrifice of the scapegoat child must be redeemed and resolved.

### Intergenerational Transfer of Unresolved Emotions

Fathers are the ones that eat sour grapes, but it is the teeth of the sons that get set on edge. (Jer 31: 29)

A child, in this sense, becomes a sacrificial lamb of past propriety when unresolved issues, pain and conflicting emotions, resentment and lack of forgiveness are constantly repeated and renewed over generations. Adults can achieve post-traumatic growth through spirituality expressed in forgiveness and gratitude (Fincham et al., 2022; Ji-yeon & Jimin, 2021). Otherwise, children are unconsciously forced to act out and perpetuate unresolved conflicts and fears. In fact, this scapegoat becomes the guardian of the whole family’s painful emotions, which can be passed down through generations. Such individuals, who are extremely sensitive to parental pain and are willing to absorb it unconditionally through their loyalty to the family, as this is the only way they can be of use to the family, are completely powerless in this role (Fairbairn, 1952). They unconsciously participate in conflicting family relationships by either resolving them or instead focusing attention on themselves.

The words of the Bible, however, bring us another painful realization, namely that *the sins of the fathers are punished up to the third and fourth generation (Exodus 20: 5–6)*. From this, we can deduce how children can actually become victims of past family conflicts, with the conflicted relationship between parents and grandparents inevitably affecting the child’s development and spiritual and psychological growth (Amato & Cheadle, 2005). The evil already committed can have consequences for future generations. Children who grow up in families where conflictual relationships, disputes, and separations are prevalent can suffer disastrous emotional consequences (Amato, 2014; Doba et al, 2022). They may develop difficulties in adapting to the demands of the outside world; in particular, they may find it difficult to form intimate partnerships later in life.

### Projective Identification as a Psychological Mechanism of Intergenerational Transfer

Family psychology explains intergenerational transfer with the concept of projective identification (Scharff & Scharff, 2000, 2014). Projective identification occurs between all members of the family. It means that an individual externalizes a state that they cannot accept within themselves by attributing that state to another (Freud, 1912). Unacceptable internal emotional states are related to experiences in a relationship. Emotions that others have not understood or that are not understood by the person themselves can become uncontrollable. Thus, the responsibility to resolve this tension involves loyal family members who, because of their integration into the family system, respond to it, accept it, and begin to behave accordingly (Scharff & Scharff, 2014). It is usually the children who take on this role, as their sense of loyalty is the only way to maintain kinship with the family in the given situation (Winnicott, 1965). The pain of the past generations is thus transferred to the descendants.

Parents who have themselves been physically punished tend to physically punish their children (Socolar et al., 2007; Straus, 1994), thus passing on the victim role to a new generation. Parents who have experienced physical punishment and are more likely to develop conduct disorder or antisocial behavior (Stuewig & McCloskey, 2005) and are more prone to stress are also less assertive and more impulsive in their responses (Straus & Mouradian, 1998), increasing the likelihood that they will react violently to their own children’s behavior. Parental pain, emotional deprivation, and psychological wounds are thus passed on through repeated dysregulated responses from one’s own childhood (Galambos & Costigan, 2003). In cases of violence, emotional dysregulation, understood as maladaptive, inflexible emotional management strategies, limited emotional expression, inability to control the timing or intensity of emotions, emotional incompetence, or inability to integrate different emotions (Hyoun et al, 2009), results in an inability to show a nonphysical response or maintain nonphysical contact when confronted with a particular physical experience. When parents confronted with their child’s inappropriate behavior are unable to regulate their emotional experience, they respond to their child in a dysregulated manner, which means they are likely to use physical force to relieve their own and their child’s physical irritation (Gross, 2013; Hyoun et al., 2009). Thus, it can be said that although the parents are the bearers of pain, it is the children who feel and experience it:* Fathers are the ones that eat sour grapes, but it is the teeth of the sons that get set on edge.*

## Redemption

From the perspective of the Christian paradigm, we might also acknowledge that the Old Testament already offers us a new hope. From this perspective, we could see that in the biblical sense neither the family nor the relationships within the family have the last word, but the redemptive grace of God.

### Sacrifice Becomes Redemption

What do you mean when you use this proverb concerning the land of Israel, saying: The fathers are the ones that eat sour grapes, but it is the teeth of the sons that get set on edge? As I live, says the Lord GOD, you are surely not going to use this proverb in Israel anymore! Behold, all souls are mine; as the soul of the Father is mine, so is the soul of the Son: the soul who sins will die. (Ezekiel 18: 2–4)

Before I formed you in the womb, I knew you; and before you came out of the womb, I sanctified you. I have appointed you a prophet to the nations. (Jeremiah 1: 5)

Can a woman forget her suckling child, that she should not have compassion on the son of her womb? Yes, they may forget, yet will I not forget you. (Isaiah 49: 15)

We can see in these words a God who sanctifies the child in all things, and requires no great sacrifice for his service. The book of Psalms also adds a significant transition in the understanding of sacrifice to God in general. The psalmist sharply criticizes the treachery and dissimulation of sinners who enumerate God’s commandments and offer thanksgiving while their actions prove otherwise. *Offerings, sacrifices and addressing God without the sincere intention of doing God’s will, will not bring salvation to sinners.* (Psalms 50: 17–22). A still deeper transition is found in Isaiah (Isaiah 1: 11). The prophet *scorns the slaughter and burnt offerings of goats* in anger. They mock God with their heinous deeds, so he exhorts them to *wash themselves, to cleanse themselves and to put away their evil deeds* (Isaiah 1: 16). Isaiah also promises a Savior in whom the Spirit of the Lord will rest and who will announce *the coming of the Messiah* (Isaiah 11: 1–9). The *servant of God,* as he is called by Isaiah (Isaiah 52: 13), who is to *replace all sacrifices and burnt offerings* (Isaiah 52: 13)*.* Despised and rejected by the people, he *indeed bears our sickness and pain;* he is wounded by our transgressions, broken in pieces for our iniquities (Isaiah 53: 3–5). Thus he embodies a propitiation for our guilt and sins by taking them upon himself. The Servant of the Lord thus becomes a sacrifice Himself and an offering for the sins of mankind. The actual process of reconciliation and redemption is thus initiated.

### Christ’s Sacrifice as an Ultimate Redemption

Isaiah’s prophecy is fulfilled in the New Testament with the sacrifice of Christ on the cross. The role of the scapegoat is thus finally confirmed in all its depth. This is not done by banishment; the scapegoat or sacrificial lamb itself brings redemption and atonement for our sins. The role of the scapegoat is thus given meaning and his suffering is confirmed. Christ’s sacrifice for the sins of all humanity is accomplished once and for all.

He doesn’t need to offer sacrifices every day, as high priests do, first for their own sins and then for the sins of the people. He did this once for all when he offered himself. (Heb 7: 27)

He did not enter by means of the blood of goats and calves; but he entered the Most Holy Place once for all by his own blood, thus obtaining eternal redemption. (Heb 9: 12–16)

This propitiatory sacrifice embraces all our painful contents, which often lead us astray. Forgiveness can be obtained in the hereafter, if only we engage in his merciful propitiation and redemption, which he has given us by his passion and resurrection. In short, He is here to heal our painful and conflicting relationships, especially with God, but also with those who separate and alienate us. Participating in the process of redemption through Christian compassion, which in practice means praying for release from old patterns of behavior, feelings, and thoughts, allows for healing redemption and resolution of conflicting, unresolved, and sinful issues that call for reconciliation. Research confirms the importance of religious coping strategies when facing adversity, and facilitate the use of reappraisal, in reducing the impact of distressing emotions on well-being. Results validate millennia-old traditional coping practices and shed light on psychological factors influencing adaptive behaviors that promote increased resilience, reduce symptoms of distress, and maintain emotional well-being (Dolcos et al, 2021).

### Preventing Physical Violence with Promoting Vulnerability and Honesty in Parenting

In the New Testament, we discover not only a radical change in the raising of children, but also a clear and unequivocal statement that a child is of the greatest value and should be respected.

Whosoever therefore shall humble himself as this little child, the same is greatest in the kingdom of heaven. And whoso shall receive one such little child in my name receiveth me. But whoso shall offend one of these little ones which believe in me, it were better for him that a millstone were hanged about his neck, and that he were drowned in the depth of the sea. (Matthew 18: 4–6)

A child also becomes a representative and symbol of how the Gospel should be concretized and materialized in the society into which Christ is introducing a revolutionary change.

And Jesus, perceiving the thought of their heart, took a child, and set him by him, And said unto them, Whosoever shall receive this child in my name receiveth me: and whosoever shall receive me receiveth him that sent me: for he that is least among you all, the same shall be great. And John answered and said, Master, we saw one casting out devils in thy name; and we forbad him, because he followeth not with us. And Jesus said unto him, Forbid him not: for he that is not against us is for us. (Luke 9: 47–50)

And whosoever shall offend one of these little ones that believe in me, it is better for him that a millstone were hanged about his neck, and he were cast into the sea. (Mark 9: 42)

But Jesus said, Suffer little children, and forbid them not, to come unto me: for of such is the kingdom of heaven. (Matthew 19: 14)

And they brought young children to him, that he should touch them: and his disciples rebuked those that brought them. But when Jesus saw it, he was much displeased, and said unto them, Suffer the little children to come unto me, and forbid them not: for of such is the kingdom of God. Verily I say unto you, Whosoever shall not receive the kingdom of God as a little child, he shall not enter therein. And he took them up in his arms, put his hands upon them, and blessed them. (Mark 10: 13–16)

Finally, we can clearly see that in the biblical text there is a radical change in the way children are brought up, not by disciplining them, which can also consist in violence, but the main task of the parents is to discover and encourage the child’s talents, creativity, and spontaneity. This is also in line with modern psychotherapy, in which parents are also called upon to discover their children’s abilities and potential.

And when he was twelve years old, they went up to Jerusalem after the custom of the feast. And when they had fulfilled the days, as they returned, the child Jesus tarried behind in Jerusalem; and Joseph and his mother knew not of it. But they, supposing him to have been in the company, went a day’s journey; and they sought him among their kinsfolk and acquaintance. And when they found him not, they turned back again to Jerusalem, seeking him. And it came to pass, that after three days they found him in the temple, sitting in the midst of the doctors, both hearing them, and asking them questions. And all that heard him were astonished at his understanding and answers. And when they saw him, they were amazed: and his mother said unto him, Son, why hast thou thus dealt with us? behold, thy father and I have sought thee sorrowing. And he said unto them, How is it that ye sought me? wist ye not that I must be about my Father’s business? And they understood not the saying which he spake unto them. (Luke 2: 42–50)

The reconciliation of the family is possible when each of its members is confronted with the scapegoat within him- or herself and so resolves to abandon his or her links with the past. God promises reconciliation and forgiveness in our actual relationships, once we decide to enter into Christ’s merciful redemptive process through sincere prayer, such forgiveness can be fundamental. Participation and faith in this process brings redemption to the scapegoat, primarily by integrating and validating his pain and freeing himself from the emotions that others have been unable to cope with. In the words of psychology, old patterns receive a new response, based on which the compulsive repetition of the affective environment of past generations is interrupted and the inner experience of both the perpetrator and the victim can be reorganized (Siegel & Solomon, 2013; Stern et al., 2010). People who experience vulnerability and honesty are able to create space for transformation in their relationships, where they can take responsibility for their own experiences without attributing their helplessness and inability to another (Firestone, 2000). This process is especially necessary in relationships with the children in the family so that no further sacrifices are made. Through their vulnerability and honesty, parents can create emotional space for their child. Parents literally form the brain of a child in a relationship, and the relationship is thus involved in the development of the child’s experience, self-regulation, self-reflection, and spirituality (Schore, 1994, 2012, 2019a, 2019b; Siegel, 2013). Through reconciliation, redemption, or the creation of a new relational experience, the child’s brain can be recreated in a different way, freed from past sins, pain, and unfulfilled desires.

## Conclusion

The child, as the most vulnerable and exposed member of the family, can play a crucial role in maintaining and changing standard patterns of behavior, emotions, and thinking, since it is the child who through his or her vulnerability, can bring the whole family to atonement. Already in the first months of a child’s life, a substantial bond is established between mother and child, which forms the basis for physical and emotional experiences through bonding and attachment with each other (Schore & Schore, 2008; Schore, 1994, 2012, 2016, 2019a, 2019b). During adolescence, these experiences develop into lasting patterns of emotional regulation, behavioral reactions and thought processes (Martin et al, 2017; Siegel, 2013). Children can thus shape their inner experience and adapt their behavior, emotions, and way of thinking to the family dynamics. In doing so, the child can take on the role of scapegoat or, especially in cases of violence, that of sacrificial lamb or victim. Parenting today is no longer understood as the subjugation of the child, but rather as an efficient integration between following the child’s needs and responding to his or her developmental and personal characteristics on the one hand, and limiting his or her behavior as well as forming his or her social engagement capacity on the other (Maccoby & Martin, 1983). Sacrifice of the child is no longer taken for granted and acceptable, and we are increasingly sensitive to any expression of violence.

In raising a child, parents also take responsibility for the consequences of their past experiences, for only in this way can they go beyond the usual patterns of response and offer new forms of relationships. In this way, parents enable their children not to have to pull teeth from the sour grapes they themselves experienced in childhood. Children are not able to control family violence, they can only adapt their mechanisms of self-regulation and self-control, which allows them to feel a sense of belonging and emotional survival (van der Kolk, 2005). An adult, on the other hand, regardless of the nature of their past experiences, may be able to take responsibility for their behavior in the present through self-control. An adult’s efficient self-control enables the regulation of painful affect without resorting to destructive behavioral mechanisms (Schore, 1994, 2019a, 2019b). This means that parents can peacefully appease the child’s maladaptive behavior so that the child is able to form a nonviolent internal regulatory system (Schore & Schore, 2008). Christ’s sacrifice on the cross is acknowledged in the family when parents face their pain and end the transference of dysregulated responses, violence, and sacrifice once and for all. Children are then no longer scapegoats or sacrificial lambs, but can also become a source of redemption, reconciliation, transformation, and development. While transforming the basic mechanisms of self-control and emotional regulation in their parenting, parents themselves can break the cycle of violence by containing, processing, and appeasing distressing physical experiences and their own regulated behavior (Finkenauer et al., 2015). In this way, they can free themselves from the role of perpetrator and the child from the role of victim. However, forgiveness and gratitude seem to be important factors for post-traumatic growth (Ji-yeon & Jimin, 2021), even in the case of physical violence in the family.

The basis of redemption lies in the creation of a new relationship through the creation of the transcendent. As we can find development and transformation in the understanding of the role of the child in the text of the Bible, we can expect such transformation and development in every family. Every relationship, especially intimate family relationships, allows for transcendence and development (Gostečnik et al., 2008). However, transformation can only occur in a deep and honest understanding of one’s emotional experience, which means that the basis for lasting relational transformation lies in changing affective perceptions of relationships (Stern et al., 2010). In the parent–child relationship, this means that parents can create the opportunity for a different experience of trust and safety. They must sacrifice their own comfort given by predictable reactions and standard emotional responses, and break the dysregulated cycle of violence *once and for all*. Or, as it is written: *He did not enter by means of the blood of goats and calves; but he entered the Most Holy Place once for all by his own blood, thus obtaining eternal redemption.* (Heb 9: 12–16).
